# Attention-Based Sequence-to-Sequence Model for Time Series Imputation

**DOI:** 10.3390/e24121798

**Published:** 2022-12-09

**Authors:** Yurui Li, Mingjing Du, Sheng He

**Affiliations:** School of Computer Science and Technology, Jiangsu Normal University, Xuzhou 221116, China

**Keywords:** deep learning, time series, missing value imputation, sequence-to-sequence, self-attention

## Abstract

Time series data are usually characterized by having missing values, high dimensionality, and large data volume. To solve the problem of high-dimensional time series with missing values, this paper proposes an attention-based sequence-to-sequence model to imputation missing values in time series (ASSM), which is a sequence-to-sequence model based on the combination of feature learning and data computation. The model consists of two parts, encoder and decoder. The encoder part is a BIGRU recurrent neural network and incorporates a self-attentive mechanism to make the model more capable of handling long-range time series; The decoder part is a GRU recurrent neural network and incorporates a cross-attentive mechanism into associate with the encoder part. The relationship weights between the generated sequences in the decoder part and the known sequences in the encoder part are calculated to achieve the purpose of focusing on the sequences with a high degree of correlation. In this paper, we conduct comparison experiments with four evaluation metrics and six models on four real datasets. The experimental results show that the model proposed in this paper outperforms the six comparative missing value interpolation algorithms.

## 1. Introduction

Time series data, which have the characteristics of high dimensionality and massive data volume, are data that are recorded and retained over time in production and life. A breakdown in equipment or incorrect human operation may result in missing data. In many industries, operational data are collected over time. From these data, we can gain experience and guidance for future research. The presence of missing values impacts the features and information extracted from the data [1], which may lead to erroneous decisions. For instance, in the medical area, there might not be any monitoring of patient blood glucose or blood pressure data, which might be thought to be needless or unexpected. Similarly, manufacturing industries do not temporarily have access to data about the air quality of their workshops. There is a risk that poor air quality assessment could result in health issues for employees or even factory safety incidents.

The following categories can be made based on the characteristics of missing data:

(1) Missing at random (MAR): Missing data in the dataset depend on the observed data.
(2) Missing completely at random (MCAR): The data are independent of external factors. The term “missing completely at random” refers to data where the missingness mechanism does not depend on the variable of interest, or any other variable, which is observed in the dataset.
(3) Neither missing at random nor missing completely at random (MNAR): The data are missing not at random if the missingness is neither MCAR nor MAR (more specifically, the data are MNAR if the missingness depends on both observed variables and the unobserved variables).

Time series preprocessing methods significantly impact the final results of missing values [2,3]. A variety of approaches have been used in the past to handle missing values, such as discarding rows containing them, substituting them with zeros, or computing the missing values using the row mean or median. Nevertheless, these techniques decrease the variance of the data, while resulting in significant bias to the covariance and correlation of the data. Deleting data would be losing the diversity of data, and there is no universal metric to evaluate the algorithm’s efficiency. For the estimation of missing values, there are two traditional methods: regression estimation and nearest neighbor estimation. Regression estimation can be divided into two categories: deterministic regression and random regression. A deterministic regression estimation uses the average of the known values in a dataset. In contrast, a random regression estimation utilizes the average of the known values combined with a random value. In nearest neighbor estimation, all known values of the nearest neighbors are averaged to impute the missing values. The accuracy of the above-mentioned approaches decreases sharply as the missing ratio increases. Typically, they can only be used when missing data are low, which limits their application. As a result, early and traditional missing-value processing approaches have steadily lost their applicability.

Most existing time series data mining methods are often developed without taking into account missing values. The performance of the methods may be significantly degraded or rendered unusable when there are missing values in the data. Research on missing values for time series is therefore required.

For time series data, data loss is a prevalent issue. Since most data analysis techniques rely on complete data [4,5], using incomplete data could lead to inaccurate results. In light of this circumstance, this paper proposes an attention-based sequence-to-sequence model to address missing values in three time series. In order to predict the missing values, our model explores data from the past and the future while also figuring out the inherent structure and regularities of the sequences.

As discussed above, several issues with missing values can be summarized as follows:

(1) Traditional methods of missing value imputation have many shortcomings, such as the difficulty in dealing with high-dimensional and large-scale time series data and the ease of modifying the internal structure.
(2) Most methods for missing-value imputation appear helpless when dealing with multivariate and variable-length data.
(3) Some existing models have no ability to capture the intrinsic information of long-range time series well enough or find the key objects of interest, thus wasting computational time and reducing model performance.

This paper proposes a sequence-to-sequence (Seq2Seq) deep learning model with an additional attention mechanism, called an attention-based sequence-to-sequence model (ASSM), to address the problems above. Further, we aim to develop an Seq2Seq model with attention mechanisms to address the limitations of existing computational and predictive models. The following are the main contributions of this paper:

(1) We propose a framework for missing value imputation in multivariate variable-length time series.
(2) Bi-directional GRU is designed as a technique for capturing forward and backward features along with imputation values of time series.
(3) The encoder–decoder structure is used to extract features from high-dimensional data to retain critical information while reducing noisy and worthless data.

The rest of the paper is structured as follows: Section 2 mainly outlines existing methods for dealing with incomplete data, Section 3 introduces some of the models and mechanisms used in our framework, Section 4 presents the proposed method, Section 5 analyzes the experimental data and experimental results, and Section 6 concludes the paper.

## 2. Related Work

Concerning the research area of missing value estimation, discarding the traditional techniques of missing value estimation, prior authors have completed some related research works. Based on their characteristics, these works can be grouped into three categories: data statistics, models, and machine learning methodologies.

Methods based on data statistics: The two main categories of missing value imputation techniques are single and multiple missing value imputation methods. There are three types of single imputation approaches: mean [6,7], median, and linear. Multivariate missing value filling methods involve chain equations with multiple variables. However, these methods for estimating missing values make the unrealistic assumption that the dataset’s missing values are uniformly and randomly distributed, which is implausible in the actual dataset. Due to the emergence of artificial intelligence, machine learning modeling techniques have taken the place of statistically based missing value padding techniques.

Methods based on the model: K-nearest neighbor (KNN), random forest imputation (miss forest), expectation maximization (EM) [8,9], linear regression (LR) [9,10], and least squares (LS) [11,12]. However, these machine learning models do not consider the relationship between various parameters. Since current estimation approaches cannot distinguish between estimated and real values from output values of computational models, the estimation of missing values may be impacted. In addition, they do not forecast future values.

Methods based on machine learning: Clustering [13,14,15,16,17], KNN [18,19], and LSTM [20,21] are used to reconstruct missing values. Due to the high dimensionality of the data, clustering typically loses the structure and informativeness of the data when used to estimate missing values. Additionally, most clustering algorithms require a known number of k cluster classes. This is typically impossible when it comes to real-time series data, and performing the labeling manually is usually time consuming. Thus, these techniques appear powerless when faced with high-dimensional data or are less applicable to real-life situations.

Deep learning models have outperformed machine learning models in domains such as sequential modeling, human detection, and medical image classification due to the rapid development of deep learning in recent years. Deep learning models have been instrumental in the fields of sequence and natural language, along with recurrent neural networks (RNN) [22], long short-term memory networks (LSTM) [23,24], gated recurrent unit (GRU) [23,25], bidirectional LSTM (BILSTM) [26], bidirectional GRU (BIGRU) [25], convolutional neural network (CNN) [27], generative adversarial networks (GAN) [28], generative adversarial imputation networks (GAIN) [29], dual-head sequence-to-sequence imputation model (Dual-SSIM) [30], and so on. Even though these models deliver superior estimation results, there are still some issues that need to be resolved. For instance, these models are difficult to build and test, require training on numerous complex parameters, and do not address missing values or prediction aspects during individual training. The work proposed in this paper is an extension of dual-SSIM. The main difference between the dual-SSIM and ours is the way they distinguish sequences. The dual-SSIM cannot focus well on the most relevant parts of the missing sequence. In contrast, this paper applies the self-attention and cross-attention mechanisms to better capture the strong correlation between time series.

## 3. Preparation

**BiGRU**: The gate recurrent unit (GRU) is a recurrent neural network (RNN) with a more straightforward structure than the long short-term memory (LSTM), has fewer parameters, and is capable of saving more time during training. Additionally, it can efficiently handle the RNNs’ long-term memory and gradient backpropagation concerns. It may choose which information to remember or forget selectively. As part of this process, only data with distinct features should be kept, and unnecessary noise or data with minimal information on the features should be eliminated. Due to the characteristics of the model, it is well suited for processing and predicting time series data. However, one issue with using GRU to model time series data is that it can only capture information from front to back and cannot learn information about the data features from back to front. Bi-GRU, a combination of forwarding and backward GRU, has been proposed as a solution to this drawback. The model is applied twice in different directions, and the results obtained in the two opposite directions are then concatenated as the final output. As a result of this method, both forward and backward time series characteristics can be captured, or alternatively, a better prediction of missing values can be made.

**Autoencoder**: An autoencoder is a kind of neural network consisting of two parts, an encoder, and a decoder [31]. The encoder can convert the input high-dimensional data x into a low-dimensional hidden vector z, where z is a compressed representation of the input data, in which the structure of the original data is completely preserved, and the most significant and influential information of the original data is preserved as well. The decoder decodes the encoded low-dimensional z into a high-dimensional x′, called the reconstruction of the data; the closer it is to the original input data x represents, the better performance of the autoencoder. The overall process can be represented by Equations (1) and (2). An autoencoder neural network can be used to extract nonlinear features more effectively than linear feature extraction methods such as PCA since it can learn from the input data.

(1) z=σ(Wx+b),

(2) $$x^{\prime }=\sigma^{\prime }\left(W^{\prime }z+b^{\prime }\right).$$

Automatic encoders can be divided into four different categories:

(1) Standard Autoencoder: It consists of three layers: the input layer, the hidden layer, and the output layer. The input layer’s neuron size |x| equals the shape of the input data, the hidden layer’s neuron size |h| < |x|, which is the dimension of the extracted features, and the output layer’s neuron size |r| = |x|.
(2) Multilayer Autoencoder: A multilayer autoencoder is established when the number of hidden layers becomes multiple, and the encoder and decoder work together to form a symmetric structure. The multilayer autoencoder is typically used for training and extracting hidden features when dealing with high-dimensional and complex data.
(3) Convolution Autoencoder: These autoencoders are typically used to convert 3D data to smaller images.
(4) Sparse Autoencoder: Typically employed for classification-related tasks, this autoencoder learns a sparse representation of the data by adding constraints to the loss function.

**Figure 1:**
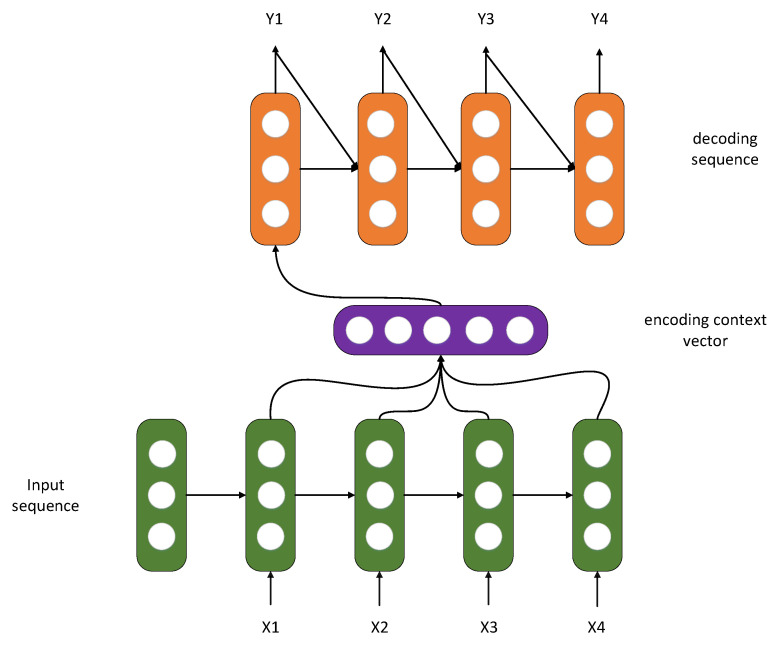
Seq2Seq model.

**Seq2Seq**: Sequence-to-sequence models refer to models that map sequence to another sequence, built based on the encoder–decoder framework, as Figure 1 shows, such as transformer. Additionally, to be used in Seq2Seq scenes, encoder–decoder structures can also be used for feature extraction and dimension reduction. The basic Seq2Seq model consists of three components: an encoder, a decoder, and a linking intermediate state vector C. During the encoding process, the encoder learns the input, which is then converted into a fixed-size state vector C and sent to the decoder. The decoder then proceeds to output the corresponding sequence by learning from the state vector C; however, basic Seq2Seq has several disadvantages, starting with the fact that the encoder encodes the input into a predetermined size state vector (hidden state), a process of “loss compression of information”. In this process of transforming vectors, the more significant the information, the greater the loss of information. The RNN model will also exhibit gradient dispersion as the sequence length increases, indicating that the sequence has been prolonged in the time dimension. The decoder cannot respond directly to more specific input information since the component of the underlying model that connects the encoder and decoder is just a fixed-size state vector. As a result of the drawbacks of basic Seq2Seq, this paper aims to improve performance by using the concepts of attention and a bidirectional encoder layer.

**Transformer**: In the majority of earlier studies, sequence modeling models such as RNN and CNN. Even though RNN models such as LSTM encode sequences can capture good long-range associations, they can only be modeled sequentially and chronologically, and the time required is relatively high. On the other hand, CNN models rely on a convolutional kernel with a windowing mechanism that moves by the step size to extract features, making them more localized and less capable of capturing long-distance correlations.

With Transformer, modeling can be performed concurrently while focusing on long-range relationships. The model encodes inputs and outputs using a global attention mechanism, enhances sequence parallelism, and enables the capture of arbitrary positional relations with a multi-head self-attention mechanism. Attention makes the model focus more on what is critical in the sequence, so it is an excellent solution to the following problems:

(1) Computing power restrictions. Recurrent neural networks (RNNs) typically increase the number of layers and the number of neurons in the hidden layer to complicate the model when a lot of information needs to be remembered, particularly long sequences, such as time sequences. Nevertheless, computational power is still the bottleneck that restricts the development of neural networks.
(2) Optimization algorithm limitations. In the face of challenges with long distances, recurrent neural networks do not have a high information memory capacity.

Transformer, the overall structure consists of four parts: the input layer, output layer, encoder, and decoder. The input layer consists of embedding and position encoding for input and target data. The input layer maps the input time series data to a vector of dimension $d_{model}$ through a fully connected network, and this step is to adopt multi-head self-attention later.

**Figure 2:**
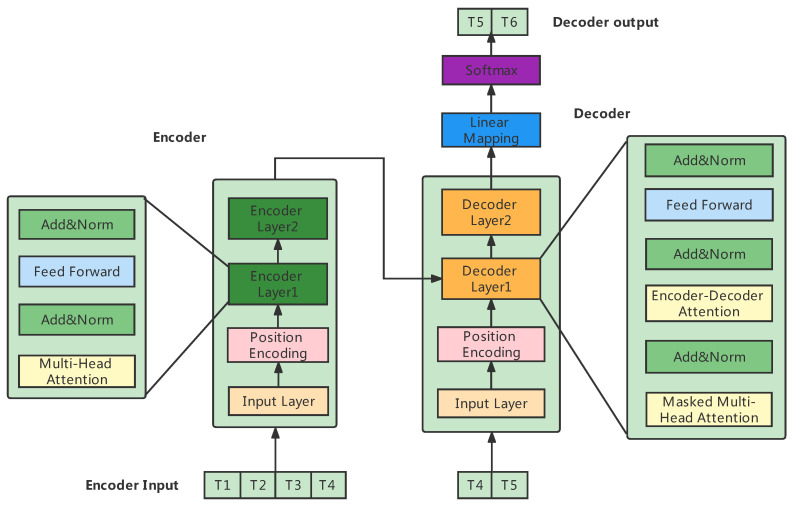
Transformer model.

The encoded vector is fed to the encoder layer with the same structure, and the number of stacked layers can be chosen freely. The encoder layer consists of *N* encoders stacked with the same structure, and each encoder can be regarded as a block composed of two sublayer structure connections. The first sublayer is a multi-head self-attention layer, a residual connection layer, and a normalized layer. The second is a fully connected feed-forward neural network, a residual connection layer, and a normalized layer. The decoder also consists of *N* blocks of the same structure, and each block is composed of three connected sublayers. The first sublayer is a multi-head self-attention layer, a residual connection layer, and a normalization layer. The second sublayer is a cross-attention layer, a residual connection layer, and a normalization layer. The third sublayer is a fully connected feed-forward neural network, a residual connection layer, and a normalization layer. The output layers include a linear layer and a SoftMax layer. The overall structure of Figure 2 is as follows.

The transformer is a framework proposed by Ashish Vaswani [32]. An upgraded version of Seq2Seq consists of an encoder and a decoder. The encoder encodes the input sequence $X=(x_{0},x_{1},x_{2},\ldots ,x_{T})$ into $H=(h_{0},h_{1},h_{2},\ldots ,h_{T})$; the decoder decodes $H=(h_{0},h_{1},h_{2},\ldots ,h_{T})$ to obtain $Y=(y_{0},y_{1},y_{2},\ldots ,y_{T})$; however, what is amazing about it is that neither the encoder nor decoder uses RNNs but replaces them with multiple attention.

Transformerś solution to the issue offered:

(1) Parallel computation and faster training. The transformer takes the place of the original RNN with attention. When the RNN is trained, the calculation of the current step depends on the hidden state of the previous step, which means that it is a sequential procedure. Each computation has to wait for the completion of the previous one before it can be started. In contrast, the training speed can be increased by using parallel computations because the transformer does not employ RNN.
(2) Establishing direct long-distance reliance. The data in the first frame of the original RNN should pass through the second, third, fourth, and fifth ⋯ frames if the first frame depends on the tenth frame. The accuracy and speed of the interaction are not guaranteed because the first frame data may have been influenced throughout the transmission procedure. On the other hand, the transformer establishes a direct dependency between any two frames, regardless of how far apart they are, because of self-attention.

**Classification of Attention**: The commonly used types are generally divided into two categories, soft attention and hard attention. Soft attention means that each word in the encoder will have a calculated probability of gaining attention, while hard attention is to find a word from the input directly that corresponds to a word in the output. Still, the time series are often linked and not just one word, so hard attention is not introduced here. Global attention means that the weight is calculated using all the encoder output, while local attention means that the weight is calculated using some encoder output. Global attention is not very different from soft attention, and local attention is between soft and hard attention. Soft attention is often seen in studies—for example, Bahdanau Attention was proposed by Bahdanau [33] and Luong presented Luong Attention [34].

The calculation process is for attention:

(1) Calculate attention weight: hidden states and encoder outputs are calculated (cosine, DNN, matrix multiplication) to obtain the score of each data point and then after SoftMax to obtain the weight result.
(2) The context vector: attention weight and encoder outputs are computed, and the weighted sum is obtained.
(3) The final result: the previous output and the context vector are concatenated, including pass shape transformation, and tanh processing; it is used as the input of the current time step.

## 4. Proposed Model

### 4.1. Notations and Definitions

**Time series**: Let $X=\left\{x_{T}^{1},\ldots ,x_{T}^{d}\right\}\in R^{d\times T}$, where X represents the dataset of time series data, d represents the number of variables in the dataset, and T is the length of the time series. $x^{i}=\left\{x_{1}^{i},\ldots ,x_{t}^{i}\right\}\in R^{T}$ denotes the ith time series data, where t≤T, the size of each time series data in a dataset can be inconsistent. $x_{t}=\left\{x_{t}^{1},\ldots ,x_{t}^{d}\right\}\in R^{d}$ represents d time vectors at time point *t*.

**Missing values in time series**: The number of input sample features is denoted as *r*, the number of output sample features is denoted as *k*, and the length of the missing sequence is denoted as *l*. If the sequence is missing from the time point *m* + 1, the missing sequence *M* is expressed as Equation (3).

(3) $$M=\left\{x_{m+1}^{i},\ldots ,x_{m+l}^{i}\right\}\in R^{i\times l},\;$$

The sequences around the missing sequence are represented by $L_{seq}$ for the data on the left and $R_{seq}$ for the data on the right, respectively. The mathematical formulas are shown in Equations (4) and (5).

(4) $$L_{seq}=\left\{x_{1},\ldots ,x_{m}\right\}\in R^{r\times m},$$

(5) $$R_{seq}=\left\{x_{m+l+1},\ldots ,x_{m+l+n}\right\}\in R^{r\times n}.$$

The variables *m* and *n* represent the length of relevant data individually.

**Model input values**: The missing data sequence is denoted as *q*, and the missing data sequence is spliced with its forward ($L_{seq}$) and backward ($R_{seq}$) sequences to form a complete input sequence X. Equation (6) is expressed as follows. The estimated missing sequence is chosen, and one of the columns is selected as the missing value imputation sequence in this paper, i.e., *k* = 1.

(6) $$X=\{L_{seq},q,R_{seq}\}$$

**Figure 3:**
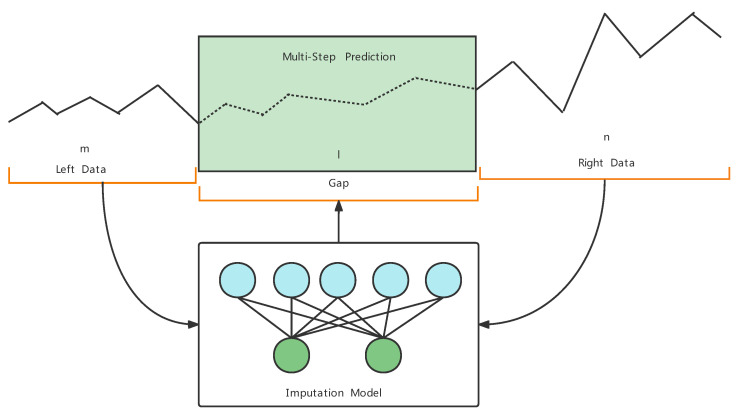
Multi-step imputation model.

**Sample generation example**: During the sample data generation, the length of the missing sequence (also known as gap size), *l*, is specified by the user. In addition, the left and right sequence lengths, *m* and *n*, can also be specified by the user, as shown in Figure 3.

### 4.2. Imputation Framework

**Figure 4:**
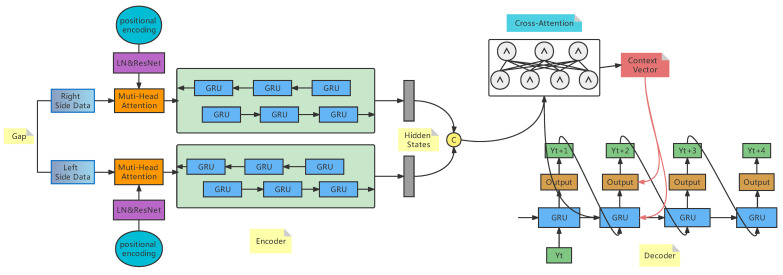
Missing value imputation model.

Seq2Seq is a sequence-to-sequence mapping process first proposed by Cho et al. [35]. A lot of work has been published since then to improve the model [36]. The application of deep learning models to sequence problems is implemented, and the more prominent area is machine translation. In the Seq2Seq model, LSTMs and GRUs are the most commonly used recurrent neural networks because they are capable of preventing gradient vanishing. The attention-added Seq2Seq model was present in [33], and the most classic paper is [34]. The GRU Seq2Seq model with attention has the advantage of fewer training parameters, faster speed, and better results, and it resolves the problem of gradient vanishing and gradient explosion during long sequence training. This improvement increases the performance of the analysis of long sequences. The overall flow chart of the GRU-Seq2Seq model with attention to missing value computation is shown in Figure 4.

### 4.3. Missing Value Prediction Model for Variable Length Time Series

#### 4.3.1. Position Encoding

When processing sequences, the self-attentive mechanism does not consider their position information. The vector operation at each position is the same and does not vary with the position. Still, there are some tasks where position information is critical, such as text generation. If position information is needed, it can be represented by position encoding [37], which usually uses sin and cos to encode the sequential information in the sequence with the following Equations (7) and (8).

(7) $$PE_{\left(pos,2i\right)}=sin\left(pos/10,000^{2i/d}\right)$$

(8) $$PE_{\left(pos,2i+1\right)}=cos\left(pos/10,000^{2i/d}\right)$$

#### 4.3.2. Multi-Head Self-Attention

Multi-head self-attention is a combination of multiple self-attention, where the input to self-attentions is a matrix X representing the sum of a data vector, approach word embedding (embedding data are the data features extracted from the original data), and position embedding. Self-attention computation usually uses the matrices *Q*, *K*, and *V*, which are obtained by linearly varying self-attention input. The typical attention mechanism uses mainly additive and multiplicative operations. The matrix add method is simpler, but considering that matrix multiplication is more mature, the form of matrix multiplication “Scaled Dot-Product Attention” is used. The self-attention mechanism can be thought of as mapping *Q* and *K* to the output, where *Q*, *K*, *V*, and the output are vectors, and the attention output is a weighted sum of *V*.

Calculate the inner product of each row vector of *Q* and *K*. The resulting matrix represents the intensity of attention between sequences divided by $\sqrt{d_{k}}$. The weights on *V* are obtained by the SoftMax activation function, where the weights of each row are added up to equal 1 (9). The inner product operations are performed using the weight matrix of *V* and the *V* matrix to obtain the self-attention result.

(9) $$Attention_{\left(Q,K,V\right)}=softmax\left(\frac{QK^{T}}{\sqrt{d_{k}}}\right)V$$

**Figure 5:**
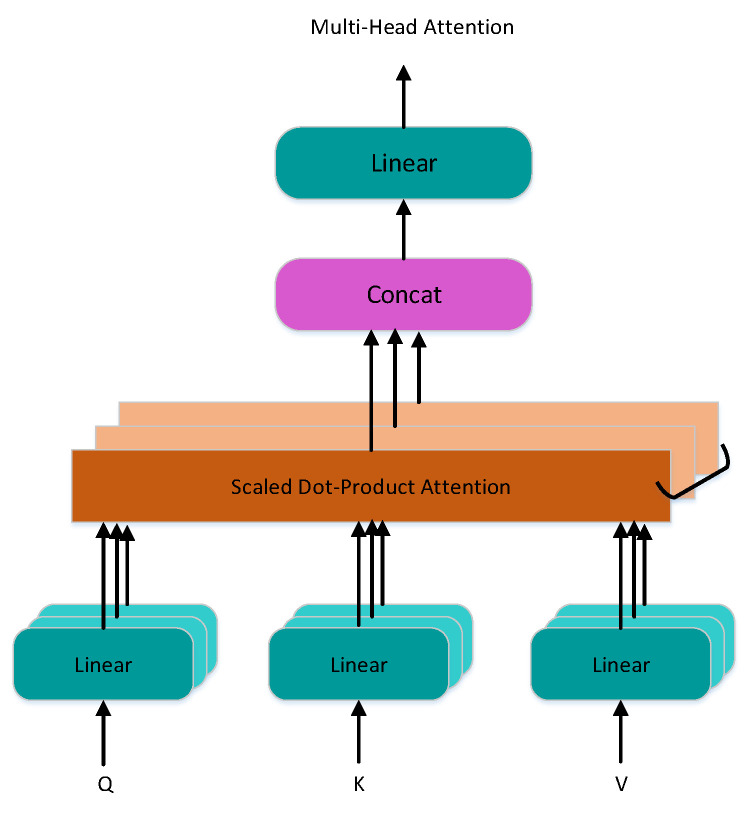
Multi-head self-attention model.

To obtain the final multi-head self-attention results, multiple self-attention results are concatenated together and linearly mapped to the same dimension as the input matrix. The computation formula is shown below (10) and (11).

(10) $$head_{i}=Attention\left(QW_{i}^{Q},KW_{i}^{K},VW_{i}^{V}\right)$$

(11) $$MultiHead_{\left(Q,K,V\right)}=Concat\left(head_{1},\ldots ,head_{h}\right)$$

where *Q*, *K*, and *V* are identical. The whole process is illustrated as Figure 5. Typically, we compute the attention functions on a set of queries simultaneously and pack them into a matrix *Q*. The keys and values are also packed into matrices *K* and *V*. In the *Q*, *K* matrix, $d_{k}$ represents the number of columns that approach the vector dimension of the matrix.

Multi-Head Attention Pseudocode: $f_{\Phi }\left(T_{l},T_{r}\right)$ is the word embedding function, where Φ is the parameter of the function. $\varphi_{\Theta }\left(T_{l},T_{r}\right)$ is the position embedding function, where Θ is the parameter. $L_{Z}\left(I+P\right)$ is the linear function, where *Z* is a parameter, and *W* stands for weight. $S(\frac{QK^{T}}{\sqrt{d_{k}}})$ is the SoftMax function, where $QK^{T}$ stands for inner product. $A_{i}$ is the result of self-attention. *n* self-attention results are stitched and then passed through a linear layer *L* to obtain the result of multi-head self-attention *U*. The multi-head attention algorithm (Algorithm 1) is as follows:
**Algorithm 1** Multi-Head Attention Algorithm.
Input: $T_{l}$, $T_{r}$ of the time series, *n* the number of attention Output: *U* the result of multi-head attention for *h* = 0 to *n* do
*I*←$f_{\Phi }\left(T_{l},T_{r}\right)$
*P*←$\varphi_{\Theta }\left(T_{l},T_{r}\right)$
Q,K,V←$L_{Z}\left(I+P\right)$
*W*←$S(\frac{QK^{T}}{\sqrt{d_{k}}})$
$A_{i}$←W·V
*U*←$L_{Z}\left(A_{1}\cup A_{2}\cup \ldots ,A_{n}\right)$
end for
return *U*


#### 4.3.3. Feed Forward Neural Network

This system is composed of two fully interconnected with the ReLU activation function. The internal structure of multi-head attention, which mainly performs matrix multiplication undergoes linear transformations, and the learning ability of linear transformations is not as strong as that of nonlinear transformations. In order to enhance learning abilities, the nonlinear mapping by activation function strengthens the part with a strong correlation and suppresses the part with a weak correlation. The formula is as follows.

(12) $$FeedForward\left(x\right)=max\left(0,w_{1}x_{1}+b_{1}\right)w_{2}+b_{2}$$

#### 4.3.4. Residual Connection and Layer Normalization

The residual connection and normalization layers are used as the components of the multi-head attention neural network and the feed forward neural network. In multi-head attention or feed forward, *x* represents the input, and MultiHeadAttention (*x*) and FeedForward (*x*) denote the output. The residual connection layer is *x* + MultiHeadAttention (*x*), and *x* + FeedForward (*x*). It is usually used during the training of multi-layer networks to focus only on the part of the current difference. The normalization layer is commonly associated with RNN structures. As a result of the normalization layer, the input of each layer of neurons will have the same mean-variance. In addition to speeding up the convergence process, it may also eliminate the influence of extreme cases on the model, resulting in a more stable network structure. The computation formula is expressed as follows.

(13) LayerNorm(x+MultiHeadAttention(x))

(14) LayerNorm(x+FeedForward(x))

#### 4.3.5. Encoder BiGRU

Data input to the bi-directional GRU layer are processed by the above layers and used as input to the BiGRU layer.

In bidirectional GRU, the hidden state is the stitching of two hidden states. BiGRU reads time series data in both directions, forward and backward, at the same time. The output has two sequences with hidden states, i.e., forward BiGRU output with the hidden states $h_{for}=\left\{\vec{h_{1}},\vec{h_{2}},\ldots ,\vec{h_{n}}\right\}$ and backward BiGRU output with the hidden states $h_{bac}=\left\{\overset{\leftarrow }{h_{1}},\overset{\leftarrow }{h_{2}},\ldots ,\overset{\leftarrow }{h_{n}}\right\}$. The variable *n* corresponds to the length of the input sequence $x_{i}$, the hidden states at time index i are concatenated as $h_{i}=\left\{\vec{h_{i}},\overset{\leftarrow }{h_{i}}\right\}$. The final output of the encoder is a sequence of hidden states $h=\{h_{1},h_{2},\ldots ,h_{n}\}$.

#### 4.3.6. Cross-Attention

The attention weight and context vector in cross-attention are calculated according to the self-attention process. The difference is that *K* and *V* are the outputs of the encoder, and *Q* is the hidden state of decoder. The outputs of the two layers of bidirectional GRU are concatenated as the outputs of the encoder. The hidden state of the last moment of the encoder is used as the initial hidden state of the decoder. The context vector and hidden state are concatenated as the result of cross-attention. The result of cross-attention and input embedding is stitched as the GRU input. The output of the decoder is the concatenated of input embedding, cross-attention result and GRU output result. Equations (15)–(17) is as follows.

(15) x=concat(attention,embedding)

(16) output=gru(x,hidden)

(17) decoder=concat(embedding,attention,output)

### 4.4. ASSM Algorithm

Ψ is the parameter associated with the encoder. *E* is the encoder output and represents the concatenated output of the two bidirectional GRU layers. $E_{K}$ is the last hidden layer state of the encoder, and it is also the initial hidden layer state *H* of the decoder. *C* is the result of cross-attention, and *Q* is the result of input embedding. θ is the relevant parameter of the decoder. *G* represents the GRU layer in the decoder. *M* represents the model encapsulation of the encoder *E* and decoder *D*. The model output is represented by *P*. According to the above introduction, the algorithm process of time series missing value imputation is shown as follows in Algorithm 2:
**Algorithm 2** Missing Time Series Imputation Algorithm.
Input: $T_{l}$, $T_{r}$ of the time series, Ψ Encoder related parameters,
θ Decoder related parameters, *n* number of iterations,
ξ overall model optimizable parameters.
Output: *P* imputation all the samples
for *i* = 1 to *n* do *E*←$E_{\Psi }\left(T_{l},T_{r}\right)$ *H*←$H=E_{K}$ *C*←$\{S\left(\frac{EH^{T}}{\sqrt{d_{k}}}\right)\cdot E\}\cup H$ *Q*←$\eta_{\theta }\left(T_{l},T_{r}\right)$ *O*←C∪Q *D*←$G_{\theta }\left(O,H\right)\cup Q\cup C$ *P*←$M_{\xi }\left(E,D\right)$
end for
return *P*


## 5. Simulation Analysis

### 5.1. Experimental Setting

**Experimental environment**: Intel(R) Core(TM) i5-1135G7@2.40GHz CPU, 24G RAM, Windows 10 operating system; programming environment is Pycharm 2021.2.2.

**Datasets**: In this paper, four datasets are selected to verify the effectiveness of the missing value imputation model. These datasets are *QLD*, *PM*25, Metro_Interstate_Traffic_Volume and ai4i2020. The details of the datasets are shown in Table 1, where *s* denotes the number of data, *k* indicates the number of variables in the dataset, *r* denotes the input data dimension, *t* denotes the number of time stamps, $t_{min}$ denotes the minimum available length, and $t_{max}$ denotes the maximum available length.

QLD: Data used in this study can be found from the Kaggle water quality dataset. Data were obtained from North Queensland, Australia, with 11 water quality monitoring stations, using a variety of sensor probes. The dataset includes nitrate concentration and water quantity. The data have been processed to remove apparent outliers and resampled so that each station may collect data from different points in time. NO3 is nitrate concentration; Temp is water temperature; Level is water level; Dayof week is week; Month is month.

PM25: This dataset includes hourly air pollutant data from 12 state-controlled air quality monitoring sites. Air quality data were obtained from the Beijing Environmental Monitoring Center. The meteorological data from each air quality site are matched with the nearest meteorological station of the China Meteorological Administration. The time is from 1 February 2010 to 31 December 2014.

Metro_Interstate_Traffic_Volume(Metro): In the dataset, *temp* is the average temperature; rain_lh is the amount of rainfall that occurs in l hour; snow_1h is the amount of snowfall that occurs in 1 hour; clouds_all is the percentage of clouds.

ai4i2020: Machine failure is the label of whether the machine failed, where there are only two values of 0 and 1. Torque [Nm] is the torque value in a normal distribution, with no negative values.

As mentioned in this paper, *d* represents the number of variables in the sample. In addition, *m* and *p* indicate the shortest and longest length of the data on the left side of the missing information, and *n* and *q* represent the shortest and longest length of the data on the right side of the missing data, respectively. The length of the output data is represented by *l*. The specific values are shown in Table 2.

A model’s parameter settings play a significant role in determining its structure. The hyperparameters of the model include the dimensionality of the input samples, the dimensionality of the output samples, the number of layers of the encoder and the decoder, the number of hidden neurons of BiGRU in the encoder, the number of hidden neurons of GRU in the decoder, the number layers of BiGRU, the number of attention, the total number of attention neurons, the number of each attention neuron, the maximum number of hidden number of LSTM units per layer, the maximum length learning rate of position encoding, dropout rate, the number of iterations, loss function, etc. The attention-GRU is implemented and optimized by using the Pytorch neural network framework. In this study, we apply a grid search over all hyperparameters for all neural network-based models. In detail, we test the numbers of layers of the encoder, decoder, and BIGRU from 1 to 3. In addition, the numbers of GRU and BIGRU units are tested in the range 25, 50, 75. For other hyperparameters, we use the recommended and classical parameter settings. The optimized hyperparameters are shown in Table 3.

### 5.2. Baseline Models

For the experiments in this paper, we compared five machine learning algorithms and one deep learning algorithm: Dual-SSIM, KNN, EM, MF, ARIMA, and Kmeans. ARIMA is one of the time series prediction analysis methods. The extensive use of missing value interpolation is Sklearn’s KNN Imputer. This method is considered an alternative to conventional interpolation. The reason for this is that it seeks to identify the K nearest neighbors of the missing data, and a value for imputation is estimated using these nearest neighbors. The expectation maximization (EM) method is a probability-based estimation method that applies to a large sample of data. The matrix factorization (MF) implemented in the fancyimpute library uses matrix decomposition methods to separate the direct matrix into low-rank elements “U” and “V”. The sparsity penalty is L1 for “U” elements and L2 for “V” elements, which are solved by gradient descent. The Kmeans algorithm divides samples into clusters and fills in the missing values based on the mean of the divided kinds. For a reasonable period, the comparison algorithms are set to default values.

### 5.3. Evaluation Metrics

In this paper, four classical machine learning evaluation metrics (18)–(21) are used to evaluate the effectiveness of missing value recovery. For time-series data, these metrics include mean absolute error (MAE), root mean square error (RMSE), symmetric mean absolute percentage error (SMAPE), and distance measure of similarity (DTW). As a general rule, the imputation effect is enhanced when all four metrics have a lower value. The formulas for the four metrics are shown below.

(18) $$MAE=\frac{1}{n}\sum_{i=1}^{n}\left|f_{i}-\hat{f_{i}}\right|$$

(19) $$RMSE=\sqrt{\frac{1}{n}\sum_{i=1}^{n}{\left(\left|f_{i}-\hat{f_{i}}\right|\right)}^{2}}$$

(20) $$SMAPE=\frac{200}{n}\sum_{i=1}^{n}\frac{\left|f_{i}-\hat{f_{i}}\right|}{\left|f_{i}\right|+\left|\hat{f_{i}}\right|}\%$$

(21) $$DTW=\min_{\pi \in A(f,\hat{f})}{\left(\sum_{(i,j)\in \pi }d{\left(f_{i},\hat{f_{j}}\right)}^{q}\right)}^{\frac{1}{q}}$$

The MAE and RMSE are two commonly used measures of variable accuracy, representing the difference between the actual and predicted values. In MAE, RMSE, and SMAPE, *n*, $f_{i}$, and $\hat{f_{i}}$ represent the sample size, the true value, and the predicted value, respectively. SMAPE is expressed as a percentage, independent of the proportion, and can be used to compare forecasts of varying proportions. This method considers not only the deviation from the true value of the predicted value but also the ratio between the deviation and the true value. It is a measurement of prediction accuracy in the statistical field and is unstable when both actual and predicted values are very close to zero. SMAPE is symmetric. In the measure, the penalty for negative error (predicted value is higher than the actual value) and positive error (actual value is higher than the predicted value) is the same. DTW is a time series similarity measure that calculates two time series of different lengths. In DTW, $A(f,\hat{f})$ is the set of all admissible paths, and $d{\left(f_{i},\hat{f_{j}}\right)}^{q}$ represents the distances at the power q.

### 5.4. Experimental Result Analysis

In order to validate the performance of the proposed model, its imputation results are evaluated with the MAE, RMSE, SMAPE, and DTW metrics. The evaluation indicators for all the compared models on four datasets are presented in Table 4. The error metrics comparison demonstrates that the ASSM model is superior to the others, such as Dual-SSIM, ARIMA, KNN, EM, MF, and Kmeans. Table 4 shows the effect of time series missing value imputation on four datasets and analyzes the imputation performance of each algorithm in detail. For each dataset, bold values indicate the highest scores obtained under the corresponding metrics.

In the four datasets, the index ranking of each model is relatively stable, as shown in Table 4. ASSM is the first, Dual-SSIM is the second, ARIMA and KNN are the third, Kmeans is the fourth, MF is the fifth, and EM is the worst. By analyzing the reason, ASSM is the SeqSeq model with added attention mechanism, and the encoder is BiGRU, which can capture data information from both forward and backward time series. By doing so, the structure and intrinsic connections between data can be captured more effectively, thereby improving the learning ability of the feature and showing a better filling effect.

**Figure 6:**
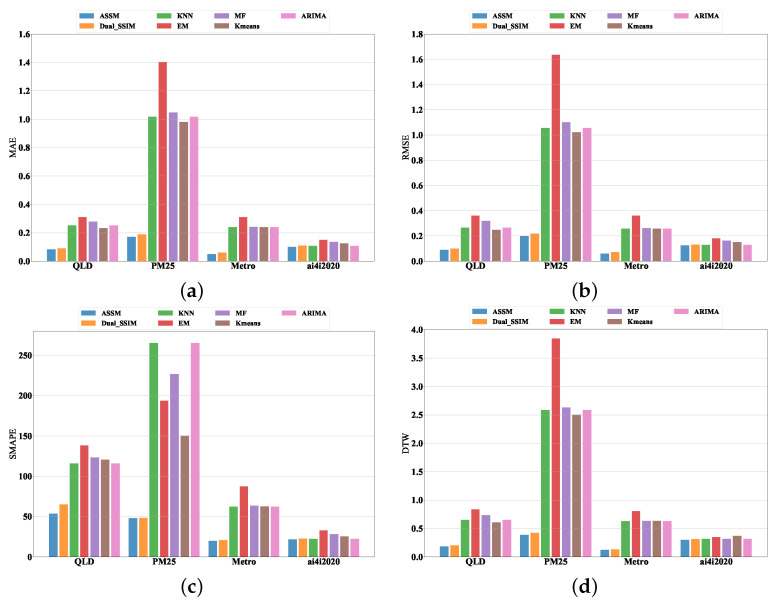
Evaluation of missing data imputation using four metrics in four datasets. (**a**) MAE of seven different methods in four datasets. (**b**) RMSE of seven different methods in four datasets. (**c**) SMAPE of seven different methods in four datasets. (**d**) DTW of seven different methods in four datasets.

During the processing of sequence data, the Seq2Seq structure is based on the unattended Dual-SSIM model and encodes a variable-length sequence X into a fixed-length vector representation C. This vector representation is then decoded into another variable length sequence Y using a decoder, where the input sequence X and the output sequence Y can differ in size. The Seq2Seq model consists of an encoder and a decoder. An encoder RNN processes the input sequence and compresses it into a vector. Its final state is a summary of the whole input sequence. Nevertheless, the encoder tends to forget some information when the input sequence is long. By adding the attention mechanism, the fixed vector C is replaced by the $c_{i}$, which is a weighted average of the encoder’s hidden states that changes with the output sequence. By employing attention, the decoder examines all the encoder states each time it updates the state, thereby preventing RNNs from forgetting their states and allowing the decoder to concentrate on the most relevant information within the encoder. Kmeans and KNN have similar effects because both are based on the calculation of sample distances and the way K values are learned. They both require artificially set values for the number of clusters and the number of nearest neighbors. The poor performance of EM may be related to the fact that EM assumes that the dataset obeys a multivariate normal distribution, which is against the actual distribution of the dataset. It is clear that ASSM provides the best performance for four metrics in all cases. For example, in the four experimental cases, ASSM achieves MAE scores of 0.083, 0.171, 0.055, and 0.105, respectively, as shown in Figure 6a. In these four cases, the best benchmark model Dual-SSIM could only achieve scores of 0.091, 0.189, 0.060, and 0.110. Similarly, in all cases, Dual-SSIM also achieves the best DTW score among all comparison methods. However, the DTW score of Dual-SSIM is still worse than that of ASSM, as shown in Figure 6d. The ASSM exhibits a remarkable performance when evaluated in the SMAPE. The ASSM achieves 53.73%, 48.09%, 19.84%, and 21.74% SMAPE scores in the four cases, see Figure 6c. The best benchmark method is Dual-SSIM in this evaluation, but its performance worse than ours.

In the four datasets QLD, PM25, Metro, and ai4i2020, the ASSM model proposed in this paper achieves the smallest value in all four metrics, approaching the best result, followed by Dual-SSIM with the second-best result. In the PM25 dataset, the ASSM model also achieves the best results in the four metrics, and Dual-SSIM comes second, while the results of the other models are nearly 10 times worse than those of the proposed model in this paper in terms of MAE measures. On the ai4i2020 dataset, the ASSM model achieves the best results in four indicators, while the Dual-SSIM model shows worse outcomes than the ARIMA and KNN models in three indicators. Dual-SSIM reached 0.110, 0.131, and 22.741 in MAE, RMSE, and SMAPE, while ARIMA and KNN achieved 0.108, 0.129, and 22.441, respectively, as shown in Table 4.

**Figure 7:**
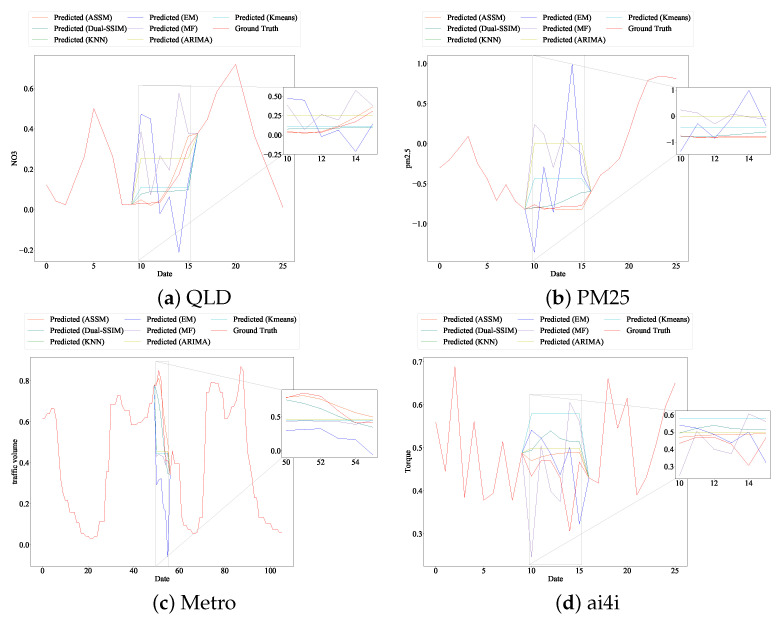
Model performance in missing sequence imputation. The solid red line is the ground truth measurements. Other colors represent the imputation results generated by different models. Twenty and fifty available data before and after the gap are used as the model’s input. Subfigures show the imputation part.

We also plot the detailed missing value-filling results for all the listed models. Figure 7a shows the performance of all the models in filling six consecutive missing NO3 measurements on the QLD dataset. In Figure 7a, the ASSM model proposed in this paper can fit the trend of the missing data well. The Dual-SSIM, Kmeans, and KNN models can approximate the overall trend of the data, while the EM, MF, and ARIMA models cannot. By providing available information before and after the missing data (red solid line), the proposed ASSM model recovers the missing data with the highest accuracy (orange solid line) with the highest accuracy. This demonstrates the effectiveness of the proposed model structure in Figure 3.

The results on the PM25 dataset see Figure 7b show that only the proposed ASSM model fits the trend and intrinsic structural pattern of the missing data well. Dual-SSIM shows a poor fit as well as the other models; for example, Dual-SSIM directly fits data with a downward trend into an overall upward trend.

We tested the memorability of the model presented in this paper on the Metro dataset. The results demonstrate that the model performed well in most cases. In the Metro dataset, the data length of the before-and-after reference data is 50, and the missing data length is 6. Based on the results of the analysis—see Figure 7c—we can conclude that the missing data are observed for a period of time as an uptrend and that the ASSM model fits an uptrend, while other models, including Dual-SSIM, ignore this uptrend and fit a downtrend.

Although the fit of each model on the ai4i dataset is not satisfactory—see Figure 7d—which may be related to the dataset, generally speaking, the ASSM model is the closest to the actual missing values of the data.

## 6. Conclusions

In this paper, we propose an Seq2Seq model with attention to fill in the missing values of time series. In terms of its overall structure, the model can be divided into two parts: encoder and decoder. The encoder employs bidirectional GRU to investigate the regularity and structure of time series from both positive and negative angles. The decoder uses cross-attention to focus on the sequence information that has a strong correlation with the missing values to be filled. The original Dual-SSIM structure does not have good differentiation of the sequences. This paper applies the self-attention and cross-attention mechanism to better capture the strong correlation between time series, strengthen the parts with strong correlation and weaken the parts with weak correlation. This significantly improves the accuracy of filling in missing values. According to experimental results on four datasets, this model outperforms the other six comparison algorithms. Experimental results report the proposed model recovers missing data series more precisely than other benchmark approaches, including Dual-SSIM, KNN, EM, MF, ARIMA, and Kmeans. Moreover, our model is able to produce steady estimation results on the four datasets, indicating that it is very promising for most time series data recovery problems.

In future work, we intend to improve further our model’s imputation accuracy in datasets with a high percentage of missing values. On the other hand, we intend to train our model using data generated from production in different domains to apply that work to real scenarios. In addition, we will combine our framework with prediction models to accurately predict time series with missing values. 

## Figures and Tables

**Table 1:** Statistical information of dataset.

Dataset	*s*	*k*	*r*	*t*	$t_{\min}$	$t_{\max}$
QLD	29,052	8	6	27,256	21,427	27,256
PM25	43,800	11	7	31,752	29,565	30,438
Metro	48,204	8	5	45,213	42,456	45,213
ai4i2020	10,000	13	11	9564	8912	9564

**Table 2:** Parameter settings for the sample generation algorithm.

Parameter	QLD	PM25	Metro	ai4i2020
*d*	6	7	5	11
*m*	10	10	50	10
*n*	10	10	50	10
*p*	10	10	50	10
*q*	10	10	50	10
*l*	6	6	6	6

**Table 3:** Parameter settings.

Hyperparameters	Value
Input dimension of Samples	*r*
Output dimension of Samples	1
Output length of Samples	6
No. of Encoder Layers	1
No. of Decoder Layers	1
No. of Hidden Units Encoder BiGRU Layers	50
No. of Hidden Units Decoder GRU Layers	50
No. of BiGRU Layers	2
No. of Attention Heads	8
No. of Hidden Units Attention Layers	512
No. of Hidden Units each Attention Heads Layers	64
Max length of position Embedding	30
Dropout Rate	0.2
Iterations	500
Learning Rate	0.005

**Table 4:** Performance of evaluated algorithms on synthetic data sets.

Datasets	Metric	ASSM	Dual-SSIM	ARIMA	KNN	EM	MF	Kmeans
QLD	MAE	**0.083**	0.091	0.252	0.252	0.310	0.279	0.232
	RMSE	**0.096**	0.103	0.265	0.265	0.361	0.320	0.248
	SMAPE	**53.732**	65.142	115.987	115.987	138.296	123.302	120.554
	DTW	**0.184**	0.203	0.650	0.650	0.834	0.735	0.608
PM25	MAE	**0.171**	0.189	1.018	1.018	1.403	1.048	0.981
	RMSE	**0.200**	0.218	1.055	1.055	1.635	1.100	1.021
	SMAPE	**48.090**	48.536	265.305	265.302	193.641	226.924	150.195
	DTW	**0.388**	0.422	2.585	2.585	3.843	2.632	2.502
Metro	MAE	**0.055**	0.060	0.239	0.239	0.310	0.242	0.240
	RMSE	**0.065**	0.072	0.257	0.257	0.360	0.261	0.258
	SMAPE	**19.840**	20.690	62.256	62.256	87.540	63.578	62.624
	DTW	**0.123**	0.131	0.630	0.630	0.805	0.632	0.633
ai4i2020	MAE	**0.105**	0.110	0.108	0.108	0.150	0.136	0.126
	RMSE	**0.125**	0.131	0.129	0.129	0.180	0.163	0.150
	SMAPE	**21.741**	22.741	22.441	22.441	32.893	28.178	25.425
	DTW	**0.0304**	0.305	0.316	0.316	0.349	0.316	0.368

## Data Availability

All datasets used for this study have been deposited in the time series imputation benchmarks repository (https://www.kaggle.com/datasets, (accessed on 8 December 2022)).
